# Neonatal nemaline myopathy associated with *NEB* variants and a favorable 3-year outcome: A case report

**DOI:** 10.1097/MD.0000000000051234

**Published:** 2026-10-23

**Authors:** Zhengxi Liu, Ya Luo, Guiping Li, Run Li, Xiaokuan Cao

**Affiliations:** a Department of Neonatology, Children’s Diagnosis and Treatment Center, The Affiliated Traditional Chinese Medicine Hospital, Southwest Medical University, Luzhou, Sichuan, China; b Department of Pediatrics, Sichuan First Hospital of Traditional Chinese Medicine, Chengdu, Sichuan, China.

**Keywords:** hypotonia, *NEB*, nemaline myopathy, neonatal respiratory failure, whole-exome sequencing

## Abstract

**Rationale:**

Nemaline myopathy (NM) is a rare congenital myopathy characterized by muscle weakness and hypotonia. Severe neonatal disease may involve respiratory failure and bulbar dysfunction. Variants in the nebulin gene (*NEB*) are a common genetic cause, but genotype–phenotype relationships and prognosis remain variable.

**Patient concerns:**

A female neonate developed cyanosis, bradycardia, respiratory distress, and hypotonia shortly after birth. She required invasive mechanical ventilation and later experienced recurrent respiratory deterioration, difficulty weaning from ventilatory support, poor feeding, vomiting and choking, weak sucking and swallowing, and diminished primitive reflexes.

**Diagnoses:**

The clinical phenotype and trio whole-exome sequencing findings supported a clinical diagnosis of NM associated with *NEB* variants. Sequencing identified a maternally inherited pathogenic frameshift variant, c.23910_23911delAG (p.Arg7970SerfsTer9), and 2 missense variants classified as variants of uncertain significance: maternally inherited c.5615C>T (p.Pro1872Leu) and paternally inherited c.19550G>A (p.Arg6517His). Muscle biopsy, allelic phasing, and functional studies were unavailable; therefore, the molecular diagnosis was not definitive.

**Interventions:**

The patient received invasive and noninvasive respiratory support, oxygen therapy, feeding and symptomatic management, serial neurological assessments, and neurological rehabilitation.

**Outcomes:**

The Neonatal Behavioral Neurological Assessment score increased from 25 on day 7 to 30 on day 14 and 36 on day 28. Respiratory function, feeding, and muscle tone gradually improved, allowing respiratory support to be withdrawn. Denver Developmental Screening Test assessments performed at 6-month intervals after discharge identified no reported developmental abnormalities. At 3 years of age, no apparent hypotonia, movement disorder, or other clinically evident motor impairment was observed.

**Lessons:**

Neonatal NM associated with *NEB* variants may present with life-threatening respiratory and bulbar dysfunction yet be followed by a favorable early developmental course. Trio whole-exome sequencing may facilitate etiological evaluation, but *NEB* variants require cautious interpretation when histopathological, phasing, and functional evidence is unavailable. A severe neonatal presentation should not be regarded as indicating an invariably poor prognosis, and continued neuromuscular, respiratory, feeding, orthopedic, and developmental surveillance remains warranted.

## 1. Introduction

Nemaline myopathy (NM) is a rare and genetically heterogeneous congenital muscle disorder. It was first described by Shy et al in 1963 after abnormal rod-like structures were identified in the skeletal muscle fibers of affected patients.^[1]^ These structures, known as nemaline bodies or nemaline rods, arise from the Z-disc and can be visualized using modified Gömöri trichrome staining or electron microscopy.^[2]^

The clinical presentation of NM varies substantially. Most affected patients present at birth or during childhood with muscle weakness and hypotonia, although the phenotypic spectrum ranges from severe neonatal disease to milder childhood- or adult-onset forms.^[2,3]^ Muscle weakness may involve the facial and bulbar muscles, neck flexors, axial muscles, proximal limbs, and respiratory muscles. Severe neonatal disease may present with respiratory failure, impaired sucking or swallowing, feeding difficulty, and reduced spontaneous movement.^[2,3]^

NM has been associated with pathogenic variants in multiple genes encoding proteins involved in the structure or regulation of the skeletal muscle thin filament and Z-disc.^[2,3]^ The nebulin gene (*NEB*), which encodes the giant sarcomeric protein nebulin, is one of the most frequently implicated genes. Most established *NEB*-related cases follow an autosomal recessive inheritance pattern.^[4]^ Because *NEB* is an exceptionally large gene with extensive normal genetic variation and no single mutational hotspot, the interpretation of individual variants can be challenging.^[4]^

The precise molecular mechanisms of *NEB*-related NM remain incompletely understood. Experimental evidence indicates that *NEB* mutations may disrupt the super-relaxed state of myosin, increase resting energy consumption in skeletal muscle fibers, and alter the muscle metabolic proteome.^[5]^ Other preclinical research suggests that NRAP may act as a disease modifier and that reducing NRAP expression could improve sarcomeric organization and skeletal muscle function in models of nebulin deficiency.^[6]^ However, no curative treatment is currently available.

This case report describes a female neonate with NM who presented with respiratory failure, repeated difficulty weaning from mechanical ventilation, hypotonia, and bulbar dysfunction and in whom 3 heterozygous *NEB* variants were identified by trio whole-exome sequencing (WES). By integrating the neonatal clinical course, genetic findings, response to supportive management and rehabilitation, and 3-year developmental follow-up, this report aims to illustrate the phenotypic variability of neonatal NM, highlight the uncertainty involved in interpreting *NEB* variants, and support cautious prognostic counseling and sustained multidisciplinary surveillance.

## 2. Case presentation

### 2.1. Patient information and family history

A female neonate was delivered by cesarean section. Complete information regarding gestational age, birth weight, indications for cesarean delivery, and prenatal ultrasonographic findings was not available in the original records. The mother had gestational diabetes mellitus. The father was reported to have glucose-6-phosphate dehydrogenase deficiency. No additional family history of congenital myopathy, neonatal hypotonia, unexplained neonatal death, or other neuromuscular disorders was documented.

### 2.2. Initial clinical presentation

At 6 minutes after birth, the neonate developed cyanosis and copious yellow foamy secretions. Her heart rate decreased to 53 beats/min. Endotracheal intubation and positive-pressure ventilation were immediately initiated. The Apgar score documented at 7 minutes was 5, with 1 point each for respiration, heart rate, skin color, laryngeal reflex, and muscle tone. Chest compressions and intramuscular epinephrine were administered, as documented in the original medical record. At 10 minutes, the Apgar score increased to 8. Despite initial resuscitation, the neonate remained mildly cyanotic and poorly responsive. Her spontaneous breathing was rapid, shallow, and irregular. Marked inspiratory retractions were observed, and oxygen saturation fluctuated between 90% and 92%. Invasive mechanical ventilation was therefore continued. Limb muscle tone was decreased, and primitive reflexes were diminished.

### 2.3. Clinical course and therapeutic interventions

The infant initially received invasive mechanical ventilation and other supportive care. After her respiratory condition improved, she was transitioned to noninvasive ventilation. On day 8, the infant again developed irritability, cyanosis, oxygen desaturation, audible laryngeal secretions, and marked inspiratory retractions. Increasing the inspired oxygen concentration did not produce substantial improvement. Endotracheal intubation was therefore repeated, and invasive mechanical ventilation was restarted. On day 9, her respiratory condition improved, allowing extubation and transition to noninvasive ventilation. Respiratory support was subsequently reduced to high-flow nasal oxygen on day 14 and oxygen supplied within the incubator on day 21.

At approximately 1 week of age, the infant demonstrated poor milk intake, recurrent vomiting and choking during feeding, weak sucking and swallowing, reduced feeding responses, hypotonia, and diminished primitive reflexes. Additional observations included a flat forehead, extension of both upper limbs during sleep, occasional staring episodes, and mildly reduced visual responsiveness. Because respiratory dysfunction, feeding impairment, and hypotonia persisted despite treatment of common neonatal conditions, the treating clinicians suspected an underlying genetic disorder. Trio WES was therefore performed using peripheral blood samples obtained from the infant and both parents. Supportive respiratory management was continued, together with serial neurological assessments and neurological rehabilitation.

### 2.4. Neurological assessment and rehabilitation

The infant underwent serial assessment using the 20-item Neonatal Behavioral Neurological Assessment (NBNA), which evaluates neonatal behavior, active muscle tone, passive muscle tone, primitive reflexes, and general neurological status.^[7]^ On day 7 after birth, the NBNA score was 25, indicating impaired neonatal neurobehavioral performance. Neurological rehabilitation was initiated. On day 14, the NBNA score increased to 30. Her milk intake and muscle tone also improved, and neurological rehabilitation was continued and intensified. On day 28, the NBNA score had increased to 36, indicating further improvement in neurological function. Rehabilitation was continued for more than 10 additional days.

### 2.5. Genetic analysis

Peripheral blood samples were collected from the infant and both parents. Trio WES was performed at Beijing MyGenostics Medical Laboratory (Beijing, China) using the WES005 Trio Whole-Exome Gene Assay V4. The testing project used the GenCap Whole-Exome Gene Capture Probe V4.0 (MyGenostics Inc.), which targeted approximately 23,000 genes across a target region of 49.11 Mb. The assay also included specially designed probes for selected intronic, intergenic, and noncoding regions associated with genes reported in the Human Gene Mutation Database. Detailed information regarding the test is presented in Table 1.

**Table 1:** Information on the trio whole-exome sequencing assay.

Project name	Capture probe	Number of genes in the target region	Length of target region
WES005: Trio Whole-Exome Gene Assay V4	GenCap Whole-Exome Gene Capture Probe V4.0	Approximately 23,000	49.11 Mb

The assay included specially designed capture probes targeting selected intronic, intergenic, and noncoding regions of *PRAMEF27*, *DISC1*, *KCNQ1*, and other genes documented in the Human Gene Mutation Database.

Mb = megabase, WES = whole-exome sequencing.

Trio WES identified 3 heterozygous variants in *NEB* (Table 2). The maternally inherited c.23910_23911delAG (p.Arg7970SerfsTer9) variant was a frameshift variant and was classified as pathogenic according to the American College of Medical Genetics and Genomics/Association for Molecular Pathology (ACMG/AMP) criteria. The maternally inherited c.5615C>T (p.Pro1872Leu) missense variant was classified as a variant of uncertain significance (VUS) and was predicted by the Rare Exome Variant Ensemble Learner (REVEL) tool to be potentially damaging (LD). The paternally inherited c.19550G>A (p.Arg6517His) missense variant was also classified as a VUS but was predicted by REVEL to be benign (B). The reported population frequencies of c.23910_23911delAG, c.5615C>T, and c.19550G>A were 0.0000083, 0.0000083, and 0.0089859, respectively. According to the original genetic testing report, c.5615C>T and c.19550G>A had not previously been reported in the literature. The ACMG/AMP classification and the REVEL prediction represent distinct levels of variant interpretation. REVEL provides in silico evidence regarding the potential functional effect of a missense variant but does not independently determine its clinical classification. Therefore, c.19550G>A (p.Arg6517His) remains a VUS despite its benign REVEL prediction. Detailed information regarding the genomic positions, transcript annotations, population frequencies, computational predictions, ACMG/AMP classifications, associated phenotypes, and parental origins of the 3 variants is presented in Table 2.

**Table 2:** Clinically relevant *NEB* variants identified by trio whole-exome sequencing.

Gene	Chromosomal location*	Transcript and exon	Nucleotide and protein change	Zygosity	Reported population frequency	REVEL prediction	ACMG classification	Associated disease/phenotype	Parental origin
*NEB*	chr2:152359891–152359893	NM_001271208; exon 167	c.23910_23911delAG (p.Arg7970SerfsTer9)	Heterozygous	0.0000083	–	Pathogenic	Nemaline myopathy type 2 (AR); congenital multiple joint contractures type 6 (AR)	Mother
*NEB*	chr2:152520210	NM_001271208; exon 45	c.5615C>T (p.Pro1872Leu)	Heterozygous	0.0000083	LD	Uncertain significance	Nemaline myopathy type 2 (AR); congenital multiple joint contractures type 6 (AR)†	Mother
*NEB*	chr2:152410418	NM_001271208; exon 126	c.19550G>A (p.Arg6517His)	Heterozygous	0.0089859	B	Uncertain significance	Nemaline myopathy type 2 (AR); congenital multiple joint contractures type 6 (AR)†	Father

Pathogenicity predictions were reported by the testing laboratory on the basis of REVEL: D, possibly damaging; LD, potentially damaging; B, benign; and –, not applicable or not available. REVEL is an in silico tool designed primarily to predict the potential functional effects of missense variants. Its predictions do not independently determine the ACMG/AMP clinical classification of a variant.

ACMG = American College of Medical Genetics and Genomics, AMP = Association for Molecular Pathology, AR = autosomal recessive, *NEB* = nebulin gene, NM = nemaline myopathy, REVEL = Rare Exome Variant Ensemble Learner.

* Genomic coordinates are presented as reported by the testing laboratory; the reference genome build was not specified.

† For the 2 variants classified as variants of uncertain significance, the disorders listed in the “Associated disease/phenotype” column represent recognized *NEB*-associated phenotypes and do not imply that either variant has been confirmed as pathogenic or causally associated with the phenotype in this patient.

The genomic coordinates are presented as reported by the testing laboratory; the reference genome build was not specified in the source report. The ACMG/AMP framework recommends classifying germline sequence variants as pathogenic, likely pathogenic, of uncertain significance, likely benign, or benign on the basis of combined population, computational, functional, segregation, and phenotype-related evidence.^[8]^ Accordingly, the 2 missense variants in this child should be described as VUSs rather than as confirmed pathogenic or benign variants.

On the basis of the neonatal clinical phenotype, including respiratory failure, difficulty weaning from mechanical ventilation, hypotonia, weak primitive reflexes, and feeding and swallowing dysfunction, together with the *NEB* findings, the treating clinicians made a clinical diagnosis of NM.

A muscle biopsy was not performed. Therefore, the presence of nemaline rods was not histopathologically demonstrated. The available records did not specify whether the 3 variants were confirmed by Sanger sequencing, whether the allelic phase of the 2 maternally inherited variants was determined, or whether RNA or other functional studies were performed.

### 2.6. Follow-up and outcomes

The child was followed at 6-month intervals after discharge. Developmental status was evaluated using the Denver Developmental Screening Test (DDST), a screening instrument assessing personal-social, fine motor-adaptive, language, and gross motor development.^[9]^ No abnormalities were identified during the reported follow-up assessments.

At the most recent follow-up, the child was 3 years old and showed no apparent hypotonia, movement disorder, or other clinically evident motor impairment. Continued long-term follow-up was planned. A detailed timeline of the patient’s clinical manifestations, diagnostic evaluation, respiratory support, therapeutic interventions, and follow-up outcomes is presented in Supplementary Table 1, Supplemental Digital Content 1.

## 3. Discussion

### 3.1. Diagnostic landscape of nemaline myopathy

This report describes a female neonate who presented with respiratory failure, repeated difficulty weaning from mechanical ventilation, hypotonia, diminished primitive reflexes, and impaired sucking and swallowing. These manifestations are compatible with the recognized neonatal presentation of NM. Nevertheless, the diagnostic evidence in this case requires cautious interpretation because neither histopathological confirmation nor conclusive evidence of 2 disease-causing *NEB* alleles was available.

Trio WES identified a maternally inherited frameshift variant, c.23910_23911delAG (p.Arg7970SerfsTer9), classified as pathogenic, together with 2 missense variants classified as VUSs. The c.5615C>T (p.Pro1872Leu) variant was maternally inherited, whereas the c.19550G>A (p.Arg6517His) variant was paternally inherited. Most established *NEB*-related NM follows an autosomal recessive inheritance pattern and requires disease-causing variants affecting both alleles.^[4]^ Because the allelic phase of the 2 maternally inherited variants was not determined and the paternally inherited variant remains a VUS, the available findings do not conclusively establish 2 pathogenic *NEB* alleles. Furthermore, the relatively high reported population frequency of p.Arg6517His and its benign REVEL prediction warrant particular caution. Computational predictions may contribute supporting evidence but do not independently establish a clinical classification.^[8]^

Additional investigations that could strengthen or clarify the molecular diagnosis include Sanger confirmation, parental phasing analysis, reassessment using updated population and disease databases, transcript analysis, functional studies, and periodic reinterpretation of the variants. Muscle biopsy was historically central to the diagnosis of NM because it enables the identification of nemaline rods. However, the number, distribution, and appearance of rods may differ among muscle fibers, biopsy sites, and *NEB*-related phenotypes, and core–rod pathological features have been described in some patients with *NEB*-related myopathy.^[2,10]^ Although avoiding an invasive procedure in a critically ill neonate may be clinically understandable, the absence of muscle histopathology limits diagnostic certainty. This case is therefore most appropriately interpreted as clinically diagnosed neonatal NM associated with *NEB* variants rather than as definitive evidence that all 3 variants caused the phenotype.

### 3.2. Comparison with typical and severe neonatal phenotypes

In a clinical study of 143 patients with NM, respiratory disease and feeding difficulties were frequently observed during the first year of life. Neonatal respiratory failure and delayed attainment of early motor milestones were associated with increased early mortality, although respiratory and feeding morbidity diminished with age in some surviving patients.^[11]^ A systematic review of pediatric NM cases similarly demonstrated substantial heterogeneity in age at presentation, genetic cause, clinical severity, systemic involvement, disease progression, and reported outcomes.^[12]^ Severe prenatal and neonatal manifestations have also been reported in genetically defined forms of NM, further illustrating the broad phenotypic spectrum of this disorder.^[13]^

The respiratory failure, recurrent ventilatory deterioration, hypotonia, weak primitive reflexes, and bulbar dysfunction observed in the present child were therefore consistent with a severe neonatal neuromuscular phenotype. However, her subsequent course was comparatively favorable. Respiratory support was gradually withdrawn, feeding and muscle tone improved, and the NBNA score increased from 25 on day 7 to 36 on day 28. At 3 years of age, no apparent hypotonia, movement disorder, or other clinically evident motor impairment was observed.

Nebulin is a giant sarcomeric protein that contributes to skeletal muscle thin-filament organization, sarcomere structure, and contractile function. Pathogenic variants in *NEB* are a major cause of NM, but genotype–phenotype correlations remain complex, and substantial clinical variability can occur among patients with variants involving the same disease-associated gene.^[4,14]^ Experimental studies of severe *NEB*-related NM have shown that pathogenic variants can lead to abnormal nebulin expression and markedly impaired skeletal muscle force generation.^[15]^ Nevertheless, the present case illustrates that marked neonatal respiratory and bulbar dysfunction may be followed by considerable early improvement. A severe neonatal presentation should therefore be interpreted carefully and should not, in isolation, be regarded as evidence of an invariably poor early childhood outcome.

### 3.3. Potential mechanisms underlying the favorable outcome

The favorable early course in this child cannot be attributed to a single established mechanism. Several explanations are biologically plausible but remain hypothetical. First, the broad genotype–phenotype variability of *NEB*-related myopathy may allow substantial functional differences among affected individuals.^[4,14]^ An allelic combination retaining partial nebulin function could theoretically contribute to a milder subsequent course; however, this possibility cannot be evaluated in the present case because variant phase, transcript effects, nebulin expression, and muscle function were not examined.

Second, respiratory and feeding morbidity may decrease with age in some surviving patients with NM.^[11]^ Maturation of respiratory control, improved respiratory muscle coordination, increased feeding efficiency, and recovery from superimposed neonatal physiological stressors could potentially contribute to clinical improvement. However, the available records do not permit these possibilities to be distinguished from one another. In particular, incomplete information regarding gestational age, birth weight, prenatal findings, and the neonatal differential diagnostic evaluation prevents determination of whether perinatal factors contributed to the initial respiratory severity.

Finally, respiratory support, feeding management, symptomatic treatment, and neurological rehabilitation may collectively have supported recovery during the neonatal period. The temporal association between these interventions and improvements in respiratory function, feeding, muscle tone, and NBNA scores is clinically noteworthy. Nevertheless, because this is a single-patient report without a comparator, the favorable outcome cannot be attributed specifically to neurological rehabilitation or any individual intervention. Improvement may have reflected supportive treatment, postnatal maturation, the natural disease course, or a combination of these factors.

### 3.4. Long-term follow-up and clinical implications

No curative treatment is currently available for NM. Management therefore focuses on respiratory support, prevention and early treatment of respiratory infections, assessment of feeding and swallowing, nutritional support, rehabilitation, preservation of joint mobility, and surveillance for scoliosis and other complications. Consensus recommendations for congenital myopathies emphasize coordinated neurological, respiratory, nutritional, gastrointestinal, orthopedic, and rehabilitation care.^[16]^

In the present case, DDST assessments performed at 6-month intervals identified no reported developmental abnormalities, and no apparent motor impairment was observed at 3 years of age. However, the DDST is a developmental screening instrument rather than a comprehensive neuromuscular assessment. A favorable result during early childhood does not exclude later respiratory muscle weakness, exercise intolerance, contractures, scoliosis, feeding problems, or other neuromuscular manifestations.

Continued multidisciplinary follow-up is therefore warranted even in the absence of overt early motor abnormalities. Follow-up should include assessment of respiratory function, muscle strength and endurance, feeding and swallowing, growth and nutritional status, orthopedic complications, and neurodevelopment. Periodic reinterpretation of the *NEB* variants should also be considered because variant classifications may change as additional population, segregation, functional, and disease-specific evidence becomes available.

### 3.5. Strengths and limitations

The principal strength of this report is the integration of a detailed neonatal clinical course, trio WES, serial neurological assessment, and 3-year developmental follow-up. The case emphasizes that an underlying congenital neuromuscular disorder should be considered when neonatal respiratory distress is accompanied by hypotonia, bulbar dysfunction, and repeated difficulty in weaning from ventilatory support. It also highlights the need for caution when counseling families about prognosis solely on the basis of severe neonatal manifestations.

Several limitations should be acknowledged. First, muscle biopsy was not performed, and nemaline rods were not histopathologically demonstrated. Second, detailed information regarding sequencing depth, target coverage, bioinformatic processing, Sanger confirmation, and variant phase was unavailable. Third, 2 of the 3 *NEB* variants were classified as VUSs, and the available genetic evidence did not conclusively establish 2 pathogenic alleles. Fourth, important perinatal information – including gestational age, birth weight, the indication for cesarean delivery, and prenatal ultrasonographic findings – was unavailable in the original records. The absence of these data limited complete characterization of the perinatal context and assessment of whether additional perinatal factors may have contributed to the severity of the initial respiratory presentation. Fifth, the records did not provide a complete account of the neonatal differential diagnostic workup. Sixth, the follow-up assessment relied mainly on developmental screening and clinical observation rather than standardized neuromuscular, respiratory, or functional outcome measures. Finally, this is a single case without a comparator; therefore, the observed improvement cannot be causally attributed to neurological rehabilitation or any other individual intervention, and the absence of apparent motor abnormalities at 3 years of age does not exclude later respiratory, orthopedic, or neuromuscular manifestations.

## Acknowledgments

The authors thank the child’s parents for their trust, cooperation, and support. The authors also thank the Affiliated Traditional Chinese Medicine Hospital of Southwest Medical University for providing the clinical platform for the patient’s treatment and follow-up.

## Author contributions

**Investigation:** Zhengxi Liu, Ya Luo, Guiping Li.

**Methodology:** Zhengxi Liu.

**Validation:** Ya Luo, Xiaokuan Cao.

**Resources:** Guiping Li, Run Li, Xiaokuan Cao.

**Supervision:** Xiaokuan Cao.

**Writing – original draft:** Ya Luo.

**Writing – review & editing:** Ya Luo, Xiaokuan Cao.


