# Supplementary material for: Neonatal nemaline myopathy associated with NEB variants and a favorable 3-year outcome: A case report
Source: Medicine (Baltimore). 2026 Oct 23;105(43):e51234. doi: 10.1097/MD.0000000000051234 (PMC13706487; doi:10.1097/MD.0000000000051234)
Supplement: Supplementary file 1 [file medi-105-e51234-s001.docx]

**Supplementary Table 1.** Timeline of the patient’s clinical manifestations, diagnostic evaluation, therapeutic interventions, and follow-up outcomes

| **Time point** | **Clinical findings, investigations, and interventions** |
| --- | --- |
| Six minutes after birth | Cyanosis, copious yellow foamy secretions, a heart rate of 53 beats/min, respiratory distress, and hypotonia were observed. Endotracheal intubation and positive-pressure ventilation were initiated. |
| Seven minutes after birth | The documented Apgar score was 5. Chest compressions and epinephrine were administered. |
| Ten minutes after birth | The Apgar score increased to 8. Persistent respiratory abnormalities necessitated invasive mechanical ventilation. |
| Day 7 | The NBNA score was 25, and neurological rehabilitation was initiated. |
| Approximately one week of age | Poor feeding, recurrent vomiting and choking, weak sucking and swallowing, hypotonia, and diminished primitive reflexes were observed. Because respiratory dysfunction, feeding impairment, and hypotonia persisted, an underlying genetic disorder was suspected, and trio WES was initiated during the neonatal hospitalization. Three heterozygous variants in NEB were subsequently identified. |
| Day 8 | Recurrent cyanosis, oxygen desaturation, and respiratory distress occurred. Endotracheal intubation was repeated, and invasive mechanical ventilation was restarted. |
| Day 9 | Respiratory status improved. The patient was extubated and transitioned to noninvasive ventilation. |
| Day 14 | The patient was transitioned to high-flow nasal oxygen, and the NBNA score increased to 30. |
| Day 21 | Oxygen was supplied within the incubator. |
| Day 28 | The NBNA score increased to 36. |
| Every six months after discharge | DDST screening showed no reported developmental abnormalities. |
| Three years of age | No apparent hypotonia, movement disorder, or other clinically evident motor impairment was observed. |

**Abbreviations:** DDST, Denver Developmental Screening Test; NBNA, Neonatal Behavioral Neurological Assessment; *NEB*, nebulin gene; WES, whole-exome sequencing.
